# IMPACT OF COVID-19 ON PEOPLE LIVING WITH HIV IN MADAGASCAR: A SARS-COV2 SERO-PREVALENCE SURVEY

**DOI:** 10.21010/Ajidv18i1.1

**Published:** 2023-10-20

**Authors:** RAKOTOMALALA Fetra Angelot, RANDRIARIMANANA Santatriniaina Dauphin, RANDRIAMANANJARA Hajanirina Nathanaella, BABIN François Xavier, RANDRIANTSARA Haja, RASAMOELINA Tahinamandranto, RAKOTO Danielle Aurore Doll, SAMISON Luc Hervé, AYOUBA Ahidjo, NERRIENET Eric

**Affiliations:** a Centre d’Infectiologie Charles Mérieux, Madagascar; b Fondation Mérieux Lyon, France; c Programme National de Lutte contre les IST-SIDA, Madagascar; d Ecole Doctorale Sciences de la Vie et de l’Environnement Université d’Antananarivo, Madagascar; e Recherches Translationnelles sur le VIH et les Maladies Infectieuses (TransVIHMI), Université de Montpellier, IRD, INSERM, France

**Keywords:** PLHIV, SARS-CoV-2, Seroprevalence, Madagascar

## Abstract

**Background:**

In Madagascar, no study has reported the impact of COVID-19 on people living with HIV (PLHIV). The present work aimed to analyze the seroprevalence of SARS-CoV-2 in Malagasy PLHIV before and during the three waves of COVID-19 pandemic.This is a retrospective study.

**Materials and Methods:**

We conducted a retrospective serological survey in PLHIV followed up for HIV viral load (VL) monitoring at the Centre d’Infectiologie Charles Mérieux Madagascar (CICM) between June 2019 and April 2022. The presence of IgM and/or IgG antibodies against SARS-CoV-2 nucleoprotein was detected using a rapid diagnostic test (COVID-PRESTO®).

**Results:**

A seroprevalence of 2.5% was found in the 877 patients tested before March 2020, compared to 25.4% (512/2,011) between March 2020 and April 2022. This seroprevalence was 21.7%, 22.3% and 60.1% after the first, second and third waves of COVID-19, respectively. We observed a marginally significant difference (p = 0.043) in SARS-CoV-2 seroprevalence between patients on highly active antiretroviral therapy (HAART) (27.5%) and those who were not (23.7%). No statistically significant difference was observed between PLHIV with undetectable HIV VL (27.4%) and the different detectable VL categories (p>0.05).

**Conclusions:**

Our data show the presence of antibodies to SARS-CoV-2 among PLHIV as early as December 2019 in Madagascar. At least 25.4% (512/2,011) of Malagasy PLHIV have been in contact with SARS-CoV-2 since March 2020. There is no significant relation between HIV-1 VL and SARS-CoV-2 seroprevalence. Additional studies with more robust assays in the general population are needed for a detailed knowledge of SARS-CoV-2 impact in Madagascar.

## Introduction

In Madagascar, the first nine cases of COVID-19 were detected on March 22^nd^, 2020 (Randremanana et al. 2021). As of November 22^nd^, 2023, 67,849 cases and 1,421 deaths related to this disease have been reported, which is probably underestimated in this country with its fragile health system. In addition, only about 6 % of the Malagasy population is fully vaccinated against COVID-19 as of February 22^nd^ 2023 (WHO 2023). A population-based study conducted in Fianarantsoa, one of the six Malagasy provinces, between February and June 2021 revealed a SARS-CoV-2 seroprevalence of 41.2% (Struck et al., 2022). Another recent study in the Malagasy blood donors registered a SARS-CoV-2 seroprevalence of 40% in 2020 (Schoenhals et al., 2021). The COVID-19 pandemic adds up to other infectious diseases conditions such as HIV/AIDS. By the end of 2021, the United Nations Programme on HIV/AIDS (UNAIDS) has estimated that there were 60,000 people living with HIV (0.2% of a total population of 27 million) in Madagascar (“UNAIDS” 2022). The objective of the present work was to determine the seroprevalence of SARS-CoV-2 among Malagasy PLHIV, which has not been documented so far.

## Materials and Methods

This is a retrospective serological analysis of PLHIV from 5 (Antananarivo, Antsiranana, Mahajanga, Toamasina and Toliara) out of 6 Malagasy provinces. The study materials were plasma samples of distinct PLHIV collected as part of their virological follow-up between June 2019 and April 2022. The plasma samples were stored at -80°C in the biobank of Centre d’Infectiologie Charles Mérieux (CICM). We detected the presence of IgM and/or IgG antibodies to the SARS-CoV-2 nucleoprotein in 10 µL of plasma of the patient using a rapid diagnostic test (COVID-PRESTO®)(Charpentier et al., 2020). In accordance with the manufacturer’s recommendations, a sample was considered SARS-CoV-2 positive if it showed an IgM or IgG or both IgM and IgG bands. Chi-square tests were performed, with a significance level of p ≤ 0.05, for i) the calculation of positivity rate per period and their 95% Confidence Intervals (95% CI); ii) the identification of the differences between SARS-CoV-2 seroprevalence in PLHIV on HAART and untreated PLHIV; iii) the identification of the differences between SARS-CoV-2 seroprevalence in PLHIV who have an RNA HIV viral load undetectable versus on PLHIV who have an RNA HIV viral load detectable; and iv) the comparison of the differences between SARS-CoV-2 seroprevalence in PLHIV for the 5 provinces of Madagascar.

## Results

The median age of the 2,888 PLWH (sex ratio: 1.2) was 32 years. Among them, 74.8% (2,161/2,888) received HAART and 82.1% (1,773/2,161) were in virological success, that is, they had a plasma HIV VL below 1,000 copies/ml.

An overall SARS-CoV-2 seroprevalence of 2.5% was found in the 877 patients tested before March 2022 compared to 21.7% of the 1056 patients tested during the first wave of the COVID-19 epidemic in Madagascar. During the second wave, 22.3% of the 770 patients tested were SARS-CoV-2 positive compared to 60.1% of the 185 patients tested during the third wave **(Table 1)**.

**Table 1:** SARS-CoV-2 seroprevalence per province in Malagasy people living with HIV during the successive waves of COVID-19. (A) Antananarivo; (B) Antsiranana; (C) Mahajanga; (D) Toamasina; (E) Toliara; (N) number of sample and (na) data not available.

Phase	Period	N tested	N positive	Prevalence per province %					Prevalence, % [95% CI]

				A	B	C	D	E	
**Pre-pandemic**	Jun 19 – Feb 20	877	22	2.1	3.6	1.9	0	4.7	2.5 [1.4 - 3.4]

**1^st^ wave**	Mar 20 – Nov 20	1,056	229	18.3	17.4	25.7	24.4	22.5	21.7 [18.9 - 23.9]

**2^nd^ wave**	Dec 20 – Jun 21	770	172	20.2	16.1	31.3	24.0	22.2	22.3 [19.3 - 25.3

**3^rd^ wave**	Nov 21 – Apr 22	185	111	60.1	na	na	na	na	60.1 [53 - 67]

We compared the SARS-CoV-2 seroprevalence in PLHIV on HAART (n = 2,161) and those who were not (n = 727). We observed a marginally significant difference between the two groups (p = 0.043) with prevalence of 27.4% and 23.6% in those on HAART and those who were not, respectively. When we stratified the prevalence of antibodies to SARS-CoV-2 by HIV-1 viral load, no statistically significant difference was observed between PLHIV with undetectable HIV RNA viral load (27.4 %) and the different categories of detectable viral loads (p>0.05).

Samples from this study were collected in 5 different provinces of Madagascar. SARS-CoV-2 seroprevalence stratified by province varied from 0% in Toamasina during the pre-pandemic period to 60.1% (112/185) in Antananarivo during the 3^rd^ wave. This distribution of seroprevalence by province is significantly different (p = 0.01) **(Table 1)**.

## Discussion

Our data first indicate the presence of SARS-CoV-2 among PLHIV, although at low frequency, below 3%, as early as December 2019 in Madagascar. This observation is in line with the hypothesis that COVID-19 was circulating at low and unnoticed level in human population already between October and November 2019 as determined by molecular clock analysis (Pekar et al., 2022). This prevalence before official COVID-19 declaration can also be due to insufficient assay performance.

Our data show that at least 25.4% (512/2,011) of Malagasy PLHIV have been in contact with SARS-CoV2 since the epidemic ignition in March 2020. A study carried out between March 2020 and November 2020 among Malagasy blood donors reported a high proportion of contamination of around 40% in this population group (Schoenhals et al., 2021). This is a rather high seroprevalence rate in this time frame of the epidemic, whatever the country considered. A plausible explanation is the quality of the assay used (first generation of SARS-CoV-2 serological assays). Else, one could also consider cluster contamination in blood donors, although this hypothesis is less likely.

Although we observed a marginal significant difference in SARS-CoV-2 seroprevalence between PLHIV on HAART and those who were not, we observed no significant relation between HIV-1 viral load and SARS-CoV-2 seroprevalence, contrarily to what was reported in a study from South Africa (Lambarey et al., 2022). This discrepancy can be explained by the sample size because the report from South Africa was from 150 patients on HAART while ours was on 2,161 PLHIV on HAART.

The explanation may also lay in other parameters/factors like covid-19 prevalence, study period, type of PLHIV targeted handled differently in the two works.

The present work also has some limitations. A first limitation of our study is the use of a single test, not a combination of tests, for our sero-survey. Another limitation is the use of a single antigen which can lead to incorrect results because of possible cross-reactions with antigens of seasonal beta-coronaviruses like OC43 and 229E (Guo et al., 2021).

Further analyses using more robust assays targeting additional SARS-CoV-2 antigens (e g, the Spike), like Luminex assay as reported elsewhere are necessary (Ayouba et al., 2020). Another perspective to this work is to perform population-based surveys as per WHO recommendation to have a global view of the pandemics in Madagascar.

## Conclusions

We report for the first time, the seroprevalence of SARS-CoV-2 in PLHIV from 5 different provinces of Madagascar. We observed a continuous increase of SARS-CoV-2 seroprevalence in PLHIV with the successive waves of COVID-19 and an inequal attack rate by province. Additional studies with more robust assays in the general population are needed for a detailed knowledge of SARS-CoV-2 impact in Madagascar.

### Ethical Considerations

This study received the approbation of the Ethical Biomedical Research Committee of Madagascar (N°107 MSANP/SG/AGMED/CNPV/CERBM).

### Funding

This project is a continuation of the support provided by Fondation Mérieux and Expertise France (Channel 1 17SANIN155 from Oct 17 to Oct 18, Channel 1 19SANIN841 from September 2019 to September 2020) and in the framework of the EVAMAD project (Channel 2 19SANIN213 from September 2020 to Sep 2023).

### Conflict of Interests

The authors declare that they have no competing interests associated with this study.

**List of Abbreviations:**
CICM: Centre d’Infectiologie Charles Mérieux Madagascar
COVID-19: Coronavirus Disease 2019
EVAMAD: Elargir l’accès aux charges virales VIH et renforce le système de santé à Madagascar
HAART: Highly Active Antiretroviral Therapy
HIV: Human Immunodeficiency Virus
IgG: Immunoglobulin G
IgM: Immunoglobulin M
PLHIV: People Living with HIV
RNA: Ribonucleic acid
SARS-CoV-2: Severe Acute Respiratory Syndrome Coronavirus 2
UNAIDS: United Nations Programme on HIV/AIDS
VL: Viral Load
WHO: World Health Organization

## References

[1] Ayouba Ahidjo, Guillaume Thaurignac, David Morquin, Edouard Tuaillon, Raisa Raulino, Antoine Nkuba, Audrey Lacroix, Nicole Vidal, Vincent Foulogne, Vincent Le Moing, Jacques Reynes, Eric Delaporte, Martine Peeters ((2020)). “Multiplex Detection and Dynamics of IgG Antibodies to SARS-CoV2 and the Highly Pathogenic Human Coronaviruses SARS-CoV and MERS-CoV”. Journal of Clinical Virology.

[2] Charpentier, Charlotte, Houria Ichou, Florence Damond, Elisabeth Bouvet, Marie-Laure Chaix, Valentine Ferré, Constance Delaugerre, Nadia Mahjoub, Lucile Larrouy, Quentin Le Hingrat, Benoit Visseaux, Vincent Mackiewicz, Diane Descamps, Nadhira Fidouh-Houhou ((2020)). “Performance Evaluation of Two SARS-CoV-2 IgG/IgM Rapid Tests (Covid-Presto and NG-Test) and One IgG Automated Immunoassay (Abbott).”. Journal of Clinical Virology.

[3] Guo Li, Yeming Wang, Liang Kang, Yongfeng Hu, Linghang Wang, Jingchuan Zhong, Hong Chen, Lili Ren, Xiaoying Gu, Geng Wang, Conghui Wang, Xiaojing Dong, Chao Wu, Lianlian Han, Ying Wang, Guohui Fan, Xiaohui Zou, Haibo Li, Jiuyang Xu, Qi Jin, Bin Cao, Jianwei Wang ((2021)). “Cross-Reactive Antibody against Human Coronavirus OC43 Spike Protein Correlates with Disease Severity in COVID-19 Patients:A Retrospective Study.”. Emerging Microbes &Infections.

[4] Lambarey, Humaira, Melissa J, Blumenthal, Abeen Chetram, Wendy Joyimbana, Lauren Jennings, Marius B. Tincho, Wendy A, Burgers, Catherine Orrell, Georgia Schäfer ((2022)). “SARS-CoV-2 Infection Is Associated with Uncontrolled HIV Viral Load in Non-Hospitalized HIV-Infected Patients from Gugulethu, South Africa.”. Viruses.

[5] Pekar, Jonathan E, Andrew Magee, Edyth Parker, Niema Moshiri, Katherine Izhikevich, Jennifer L, Havens, Karthik Gangavarapu, Lorena Mariana Malpica Serrano, Alexander Crits-Christoph, Nathaniel L, Matteson, Mark Zeller, Joshua I, Levy, Jade C, Wang, Scott Hughes, Jungmin Lee, Heedo Park, Man-Seong Park, Katherine Ching Zi Yan, Raymond Tzer Pin Lin, Mohd Noor Mat Isa, Yusuf Muhammad Noor, Tetyana I, Vasylyeva, Robert F, Garry, Edward C, Holmes, Andrew Rambaut, Marc A, Suchard, Kristian G. Andersen, Michael Worobey, Joel O. Wertheim ((2022)). “The Molecular Epidemiology of Multiple Zoonotic Origins of SARS-CoV-2.”. Science.

[6] Rindra Vatosoa Randremanana, Soa-Fy Andriamandimby, Jean Marius Rakotondramanga, Norosoa Harline Razanajatovo, Reziky Tiandraza Mangahasimbola, Tsiry Hasina Randriambolamanantsoa, Hafaliana Christian Ranaivoson, Harinirina Aina Rabemananjara, Iony Razanajatovo, Richter Razafindratsimandresy, Joelinotahiana Hasina Rabarison, Cara E. Brook, Fanjasoa Rakotomanana, Roger Mario Rabetombosoa, Helisoa Razafimanjato, Vida Ahyong, Vololoniaina Raharinosy, Vaomalala Raharimanga, Sandratana Jonhson Raharinantoanina, Mirella Malala Randrianarisoa, Barivola Bernardson, Laurence Randrianasolo, Léa Bricette Nirina Randriamampionona, Cristina M. Tato, Joseph L. DeRisi, Philippe Dussart, Manuela Christophère Vololoniaina, Fidiniaina Mamy Randriatsarafara, Zely Arivelo Randriamanantany, Jean-Michel Heraud ((2021)). “The COVID-19 Epidemic in Madagascar:Clinical Description and Laboratory Results of the First Wave, March-September 2020.”. Influenza and Other Respiratory Viruses.

[7] Schoenhals, Matthieu, Niry Rabenindrina, Jean Marius Rakotondramanga, Philippe Dussart, Rindra Randremanana, Jean-Michel Heraud, Soa Fy Andriamandimby, Paquerette Hanitriniala Sahondranirina, Manuela Christophère Andriamahatana Vololoniaina, Fidiniana Mamy Randriatsarafara, Voahangy Rasolofo, Zely Arivelo Randriamanantany, AndréSpiegel ((2021)). “SARS-CoV-2 Antibody Seroprevalence Follow-up in Malagasy Blood Donors during the 2020 COVID-19 Epidemic.”. EBioMedicine.

[8] Struck, Nicole S, Eva Lorenz, Christina Deschermeier, Daniel Eibach, Jenny Kettenbeil, Wibke Loag, Steven A. Brieger, Anna M. Ginsbach, Christian Obirikorang, Oumou Maiga-Ascofare, Yaw Adu Sarkodie, Eric Ebenezer Amprofi Boham, Evans Asamoah Adu, Gracelyn Asare, Amos Amoako-Adusei, Alfred Yawson, Alexander Owusu Boakye, James Deke, Nana Safi Almoustapha, Louis Adu-Amoah, Ibrahim Kwaku Duah, Thierry A, Ouedraogo, Valentin Boudo, Ben Rushton, Christa Ehmen, Daniela Fusco, Leonard Gunga, Dominik Benke, Yannick Höppner, Zaraniaina Tahiry Rasolojaona, Tahinamandranto Rasamoelina, Rivo A. Rakotoarivelo, Raphael Rakotozandrindrainy, Boubacar Coulibaly, Ali Sié, Anthony Afum-Adjei Awuah, John H. Amuasi, Aurélia Souares, Jürgen May ((2022)). “High Seroprevalence of SARS-CoV-2 in Burkina-Faso, Ghana and Madagascar in 2021:A Population-Based Study”. BMC Public Health.

[9] “UNAIDS” ((2022)). Country Factsheets MADAGASCAR 2021.

[10] WHO ((2023)). “Madagascar:WHO Coronavirus Disease (COVID-19) Dashboard With Vaccination Data.”World Health Organization.

